# Assay Development for Metal-Dependent Enzymes—Influence of Reaction Buffers on Activities and Kinetic Characteristics

**DOI:** 10.1021/acsomega.3c02835

**Published:** 2023-10-18

**Authors:** Natalia Forero, Chengsong Liu, Sami George Sabbah, Michele C. Loewen, Trent Chunzhong Yang

**Affiliations:** † Department of Chemistry and Biomolecular Sciences, University of Ottawa, Ottawa K1N 6N5, Canada; ‡ Aquatic and Crop Resource Development Research Centre, National Research Council, Ottawa K1A 0R6, Canada; § Department of Medicine, University of Ottawa, OttawaK1N 6N5, Canada

## Abstract

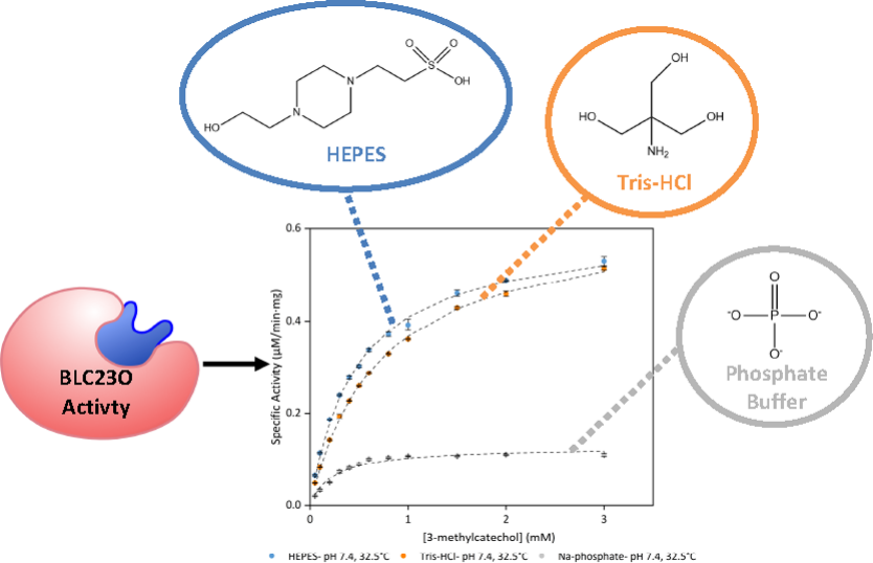

Buffers are often thought of as innocuous components of a reaction, with the sole task of maintaining the pH of a system. However, studies had shown that this is not always the case. Common buffers used in biochemical research, such as Tris (hydroxymethyl) aminomethane hydrochloride (Tris-HCl), can chelate metal ions and may thus affect the activity of metalloenzymes, which are enzymes that require metal ions for enhanced catalysis. To determine whether enzyme activity is influenced by buffer identity, the activity of three enzymes (BLC23O, *Ro*1,2-CTD, and trypsin) was comparatively characterized in *N*-2- hydroxyethylpiperazine-*N*′-2-ethanesulfonic acid (HEPES), Tris-HCl, and sodium phosphate buffer. The pH and temperature optima of BLC23O, a Mn^2+^-dependent dioxygenase, were first identified, and then the metal ion dissociation constant (*K*_d_) was determined in the three buffer systems. It was observed that BLC23O exhibited different *K*_d_ values depending on the buffer, with the lowest (1.49 ± 0.05 μM) recorded in HEPES under the optimal set of conditions (pH 7.6 and 32.5 °C). Likewise, the kinetic parameters obtained varied depending on the buffer, with HEPES (pH 7.6) yielding overall the greatest catalytic efficiency and turnover number (*k*_cat_ = 0.45 ± 0.01 s^–1^; *k*_cat_/*K*_m_ = 0.84 ± 0.02 mM^–1^ s^–1^). To corroborate findings, the characterization of Fe^3+^-dependent *Ro*1,2-CTD was performed, resulting in different kinetic constants depending on the buffer (*K*_m (HEPES, Tris-HCl, and Na-phosphate)_ = 1.80, 6.93, and 3.64 μM; *k*_cat__(HEPES, Tris-HCl, and Na-phosphate)_ = 0.64, 1.14, and 1.01 s^–1^; k_cat_/*K*_m__(HEPES, Tris-HCl, and Na-phosphate)_= 0.36, 0.17, and 0.28 μM^–1^ s^–1^). In order to determine whether buffer identity influenced the enzymatic activity of nonmetalloenzymes alike, the characterization of trypsin was also carried out. Contrary to the previous results, trypsin yielded comparable kinetic parameters independent of the buffer (K_m (HEPES, Tris-HCl, and Na-Phosphate)_ = 3.14, 3.07, and 2.91 mM; k_cat__(HEPES, Tris-HCl, and Na-phosphate)_ = 1.51, 1.47, and 1.53 s^–1^; kcat/*K*_m (HEPES, Tris-HCl, and Na-phosphate)_ = 0.48, 0.48, and 0.52 mM^–1^ s^–1^). These results showed that the activity of tested metalloenzymes was impacted by different buffers. While selected buffers did not influence the tested nonmetalloenzyme activity, other research had shown impacts of buffers on other enzyme activities. As a result, we suggest that buffer selection be optimized for any new enzymes such that the results from one lab to another can be accurately compared.

## Introduction

**Figure 1:**
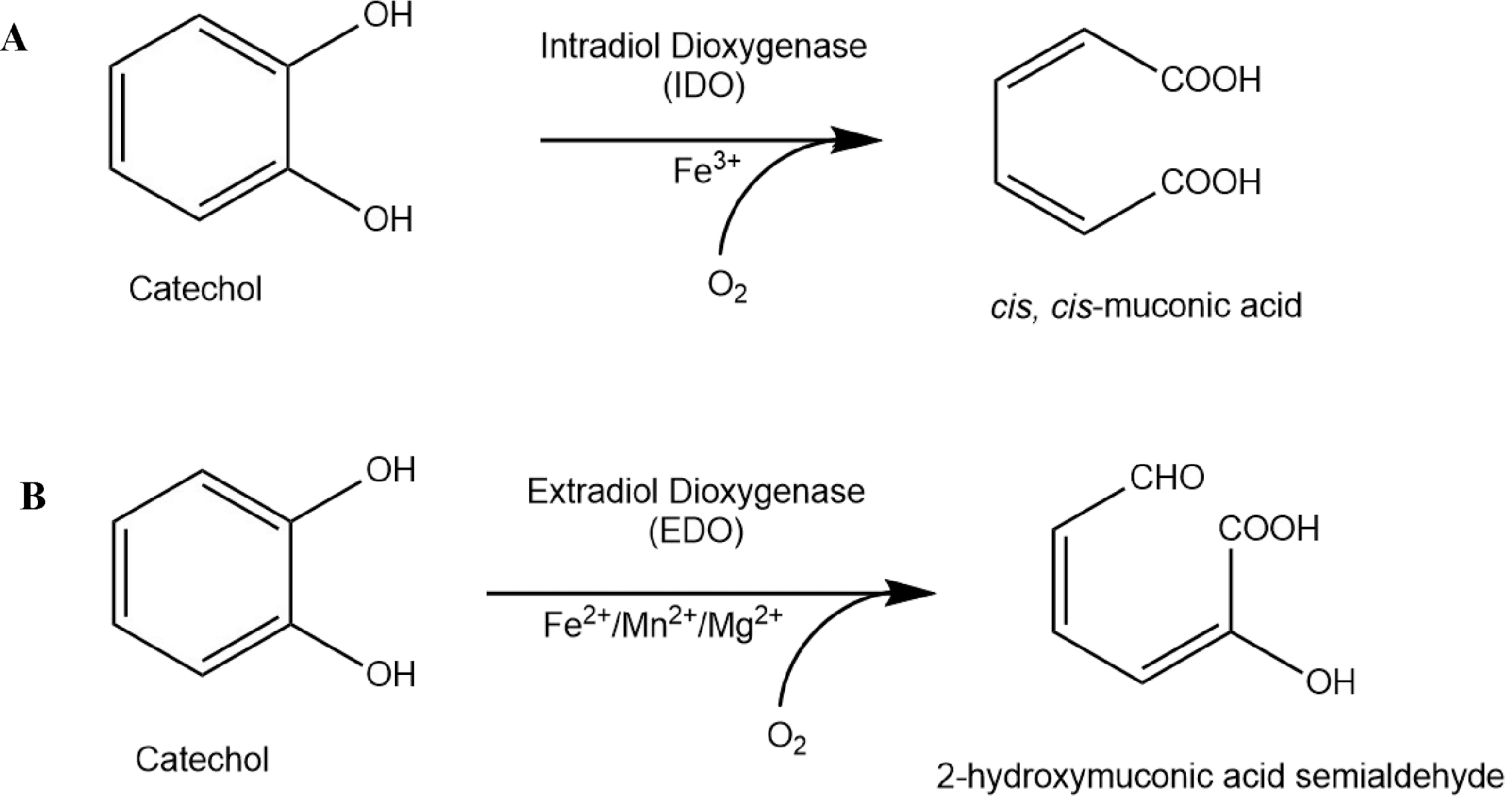
Reactions catalyzed by intradiol (A) and extradiol (B) dioxygenases. Image was generated using ChemDraw.

Buffers are integral components of biological systems. They keep the pH of a system stable around a target pH by neutralizing small additions of acid or base, therefore helping maintain homeostasis.^1^ Biomacromolecules, such as enzymes, function only within a narrow pH range. Deviation in pH can lead to changes in the enzyme structure, thereby affecting enzyme activity and stability. Given that extreme acidic or caustic pH may result in the denaturation of the enzyme,^2^ buffers impede denaturation by preventing drastic pH changes in a system. Although buffers are traditionally considered to be chemically inert in reactions, this is an incorrect assumption that may lead to the misinterpretation of results.^3^ A major challenge faced when using carboxylic acid, inorganic, and primary amine buffers is their ability to chelate metal ions^4^ and thus potentially influence the activity of metalloenzymes.^3^ Metalloenzymes are enzymes that utilize metal ion cofactors for enhanced catalysis and/or greater structural stability.^5^ About one-third of all enzymes are classified as metalloenzymes, which are diverse in both enzyme structure and function.^6^ Among these, dioxygenases perform a key step in the degradation of aromatic compounds by incorporating diatomic oxygen into the aromatic ring of catechols and derivatives, followed by ring cleavage^7^ (Figure 1).

While intradiol dioxygenases (IDOs) cleave in-between vicinal hydroxyl groups and are often Fe^3+^-dependent (Figure 1A), extradiol dioxygenases (EDOs) cleave the C–C bond next to one of the two vicinal hydroxyl groups and are usually Fe^2+^ dependent^8^ (Figure 1B). However, there are reports of magnesium-^9^ and manganese-^10−12^ dependent EDOs. Recently, our laboratory has identified a novel catechol 2,3 dioxygenase (C23O) from *Bacillus ligniniphilus* sp. L1 (BLC23O), which is a Mn^2+^-dependent EDO that has high substrate affinity for alkylated catechols (3-methylcatechol/3-ethylcatechol) in sodium phosphate buffer.^13,14^ In comparing BLC23O activities with other EDOs, particularly the other Mn^2+^-dependent EDOs, we have found that different buffers were used for the kinetic assays: 50 mM Tris-HCl (pH 7.5) was used for Mn^2+^-dependent EDO from *Bacillus* sp. JF8,^10^ KP_i_ buffer (pH 8.0) containing 0.75 mM 3,4-DHPA was used for Mn^2+^-dependent EDO from *Arthrobacter globiformis* CM-2,^11^ and potassium phosphate buffer (pH 7.5) was used for Mn^2+^-dependent EDO from *Brevibacillus brevis*.^12^ We reasoned that these enzyme activities cannot be reliably compared solely based on the reported numbers when different buffers are used. To investigate whether this is the case and if buffer identity influences enzyme characterization, we have compared the BLC23O activities in several reaction buffers.

Three buffers have been identified within the optimal pH range of BLC23O, namely, *N*-2- hydroxyethylpiperazine-*N*′-2-ethanesulfonic acid (HEPES), Tris (hydroxymethyl) aminomethane hydrochloride (Tris-HCl), and sodium phosphate. HEPES is a zwitterionic buffer initially proposed by Good et al.^15^ to replace conventional buffers used in biochemical research. It is used in enzyme characterization because of its physiological buffering range and low metal-binding constant.^2,16−18^ This makes it particularly useful in the characterization of metal-dependent enzymes. Tris-HCl is a buffer also frequently used in experimental assays involving biological systems for its physiological buffering capacity (7.0–9.0, p*K*_a_ = 8.1).^19^ However, Tris-HCl has an amino group that can partake in reactions when deprotonated^3^ and has been shown to interfere with the enzymatic activity of certain metalloenzymes.^20−22^ A recent complexation study of buffer/metal ion has shown that Tris-HCl interacts weakly with Mn^2+^ and interacts strongly with both cupric and lead ions.^18^ Lastly, sodium phosphate is an inorganic buffer usually used in the characterization of enzymes because it mimics components of the extracellular environment, and its pH changes very little with temperature. However, phosphate buffer has been found to interact and precipitate with certain metal ions,^23^ such as Ca^2+^. Therefore, phosphate buffer can potentially inhibit some metalloprotein-catalyzed reactions.^15^

Given that metal-dependent enzymes bind metal ions at catalytic centers for enhanced catalysis, we have presumed that when equal concentrations of metals were added, enzyme activities may change in different buffers at the same pH, due to the potentially different interactions between buffer chemicals and metals. Literature research^3,18,24^ has shown very limited reports that only investigate buffer impact on metal ions and overlook buffer impact on enzyme activities. Therefore, the purpose of this present study is to investigate the importance of buffer selection in assays involving metal-dependent enzymes to guide future assay development for metal-dependent enzymes. To this end, the BLC23O enzyme has been first characterized in the three buffer systems aforementioned and the kinetic parameters obtained have been compared across buffers. To corroborate the observations obtained with BLC23O, two other enzymes are also investigated: an Fe^3+^-dependent intradiol 1,2 catechol dioxygenase from *Rhodococcus opacus* (*Ro*1,2-CTD) and a nonmetalloenzyme, trypsin, from the bovine pancreas. To the best of our knowledge, this is the first study investigating the impact of reaction buffer on the reaction kinetics of metal-dependent enzymes.

## Experimental Section

### Chemicals/Reagents

Substrates (3-methylcatechol and *N*_*α*_-Benzoyl-l-arginine 4-nitroanilide hydrochloride), buffer salts (HEPES, Tris-HCl, and Na_2_HPO_4_/NaH_2_PO_4_), and metal salts (MnCl_2_·4H_2_O) were obtained from Sigma-Aldrich (St. Louis, MO, United States).

### BLC23O Expression and Cell Extract Preparation

A starter culture of LB media and *Escherichia colis* cells containing a plasmid with the BLC23O gene was used to inoculate fresh LB media at 37 °C with 50 μg/mL kanamycin. Cells were grown until the OD (600 nm) reached 0.5–0.6, after which 0.2 mM isopropyl β-d-1-thiogalactopyranoside (IPTG) was added to induce protein expression. The culture was placed under a cold shock at 4 °C for 20 min and then shaken at 200 rpm under 16 °C overnight. After incubation, the cells were collected by centrifugation at 3200*g* for 20 min at 4 °C. The clear supernatant was discarded, and the cell pellet was resuspended in lysis buffer containing 50 mM Na_2_HPO_4_, 300 mM NaCl, and 10 mM imidazole and supplemented with 0.1 mM phenylmethylsulfonyl fluoride (PMSF), 3 U/mL benzonase, and 1.0 mg/mL lysozyme. After resuspension, the cells were disrupted through sonication (10 cycles of 20-s bursts and 20-s cooling period) using a Sonifier cell disruptor 350 sonicator (Branson Ultrasonics, Brookfield, CT, United States). Cell debris was collected through centrifugation at 3200*g* and 4 °C for 20 min, and the supernatant containing BLC23O was separated and stored at −20 °C.

### BLC23O Two-Step Purification and Protein Determination

Purification of BLC23O was achieved in two steps: an affinity chromatography step and a size exclusion chromatography step. Ni-NTA resin (Qiagen, Hilden, Germany) was used for the affinity purification step, in which BLC23O with a 6xHis-tag binds to the Ni-NTA resin under a low concentration of imidazole (10 mM) solution and was eluted with a high concentration of imidazole (300 mM) solution. The enzyme was then further purified through size-exclusion chromatography on an FPLC system (UPC-900 ÄKTA, Amersham Pharmacia Biotech, Amersham, United Kingdom) using a HiLoad 16/60 Superdex 200 prep grade column (Cytiva, Malborough, MA, United States). The buffer used for FPLC was composed of 10 mM Tris-HCl and 150 mM NaCl. Following purification, SDS-PAGE was performed to identify the fractions that contain the target enzyme, as well as to determine purity (See Figure S1). The protein sample was then buffer exchanged (10 kDa MWCO) and concentrated to prepare a protein stock in 10 mM Tris-HCl and at pH 7.4, which was stored at 4 °C. Protein concentration was determined using the Bradford protein assay^25^ with bovine serum albumin (BSA) as the standard (See Figure S2).

### BLC23O Enzyme Assays

Enzyme activity was assayed spectrophotometrically by monitoring product formation through an increase in absorbance at 388 nm.^26^ 250 μL reactions containing 18.7 μg/mL, 1 mM 3-methylcatechol, and 50 mM of the respective buffers were performed in triplicate in a 96-well microplate and were initiated with the addition of substrate. The plate was monitored for 60 min, reading on every 30 s interval using the spectrophotometer SpectraMax M5^e^ (Molecular Devices, San Jose, CA, United States). For reactions requiring a specific temperature, the microplate chamber was adjusted to the target temperature, and the microplate with reagents was incubated for 15 min prior to the start of the reaction. The slope of the initial linear portion of the kinetic curve was corrected against the blank and used to determine specific activity (ε_2-hydroxy-6-oxo-2,4-heptadienoic acid_^26^ = 13,800 M^–1^cm^–1^; *M*_WBLC23O_= 31.824 kDa). Specific activity was defined as the amount of product formed in μM per minute per milligram of enzyme.

### BLC23O pH and Temperature Optimization

For pH optimization, a range of eight different pH values were selected for each buffer according to their corresponding buffering capacity. With HEPES, the pH range was chosen from 6.8 to 8.2 with an interval of 0.2. With Tris-HCl, the pH range chosen was from 7.2 to 8.6. Lastly, with the sodium phosphate buffer, the pH range chosen was from 6.6 to 7.0. Reactions were performed as described in the BLC23O enzyme assays and at 32.5 °C. For temperature optimization, reactions were performed as described in the BLC23O enzyme assays and at the optimal pH for each buffer. The temperature range tested was from 25 to 45 °C, with a 2.5 °C interval.

### Determination of BLC23O Metal Ion Dissociation Constant (*K*_d_)

Reactions were performed as described in the BCL23O enzymatic assay, with the Mn^2+^ concentration varying from 0.5 to 50 μM in HEPES and Tris-HCl. In phosphate buffer, the Mn^2+^ concentrations were from 0.5 to 200 μM. The reactions were performed under comparable conditions in all three buffers and under the established optimal conditions for each buffer: in HEPES at pH 7.6 and 32.5 °C, in Tris-HCl at pH 7.4 and 32.5 °C, and in sodium phosphate buffer at pH 7.2 and 30 °C. Data analysis was performed as outlined in the enzymatic assays section, with the addition that the specific activity curve was modeled using the Solver add-in from Microsoft Excel (Redmond, WA, United States) to solve for the kinetic parameter *K*_d_.^27^

### BLC23O Michaelis–Menten Kinetic Assays

Reactions were performed as described in the BLC23O enzymatic assays section, with the exception that 3-methylcatechol concentration varied between 0.05 and 3 mM. Kinetic assays were performed under optimal and comparable (pH 7.4 and 32.5 °C) conditions in each buffer, and the Solver add-in from Microsoft Excel was used to model the kinetic curve for *K*_m_ and *V*_max_.

### Recombinant Expression and Purification of *Ro*1,2-CTD

A gene encoding the *Ro*1,2-CTD (also known as pyrogallol dioxygenase; GenBank ID: CAA67941) was synthesized (Biobasics) and cloned into the pQE80L vector (T5 promoter and ampicillin resistant) inserted between the *Bam*HI and *Hin*dIII restriction sites, encoding an N-terminal His-tag. The resulting pQE80L-*Ro*1,2-CTD expression construct was transformed into *E. colis* BL21 (DE3) cells. Recombinant wild-type *Ro*1,2-CTD was produced using an Eppendorf New Brunswick BioFlo/Celligen 5-L Bioreactor, with Terrific Broth (per L: 24 g yeast extract, 20 g tryptone, 4 mL glycerol, 100 mL phosphate buffer 0.17 M KH_2_PO_4_, 0.72 M K_2_HPO_4_), 100 mg/mL ampicillin, and 10 μL/L culture of antifoam. The temperature was set to 37 °C, agitation level was set to 150 rpm, and dissolved oxygen minimum was set to 30%. Following inoculation with an overnight culture (10 mL/L culture) of *E. colis* BL21 (DE3) pQE80L-*Ro*1,2-CTD, growth was monitored until an OD_600_ of 0.4 was achieved, at which time the temperature was turned down to 20 °C and growth further monitored until an OD_600_ of 0.6 at which point IPTG was added to a final concentration of 0.7 mM. Following 18 h of additional fermentation, the cells were harvested by centrifugation at 3250*g* for 30 min at 4 °C and obtained cell pellets were frozen at −20 °C. When ready to proceed, frozen cell pellets were resuspended at a ratio of 1 mL lysis buffer (50 mM Tris, pH 8.0, 300 mM NaCl, 10 mM imidazole, 0.1 mM PMSF, 3 U/mL benzonase, and 1 mg/mL lysozyme) per 0.1 g of cell pellet (wet weight) and lysed by sonication (60 cycles: 25 s each at 30% duty and 40 output power, with 30 s cooling periods; Vibra-Cell). The obtained lysate was clarified by centrifugation at 17,000*g* for 30 min at 4 °C. Subsequent two-step purification was exactly as described for BLC23O above.

### *Ro*1,2-CTD Kinetic Assays

Enzymatic assays for the *Ro*1,2-CTD enzyme were 400 μL each, containing 50 mM of the respective buffers at pH 7.2,^28^ 1.37 μg/mL *Ro*1,2-CTD, and increasing concentrations of 3-methylcatechol (from 4 to 500 μM). The reactions were performed in triplicate, along with a blank, and these were monitored at 260 nm for a period of 30 min and read at 15 s interval. The absorbance was corrected against the blank, and the slope of the linear region was used to calculate specific activity (ε_2-methylmuconic acid_^29^ = 18,000 M^–1^cm^–1^; *M*_W Ro1,2-CTD_= 32.094 kDa). Kinetic analysis was performed using the Solver add-in from Excel to calculate kinetic parameters.

### Trypsin Kinetic Assays

Kinetic assays for trypsin (T1426, Sigma, St. Louis, MO, United States) were performed as outlined by Silva et al.^30^ Reactions were each 250 μL containing 100 mM of the appropriate buffer at pH 8.0, 20 μg/mL trypsin in 1 mM HCl, and increasing concentrations of the substrate *N*_*α*_-benzoyl-l-arginine 4-nitroanilide hydrochloride (BApNA). BApNA concentrations ranged from 0.1 to 15 mM and were prepared in DMSO. Reactions were performed in triplicate along with a blank. Absorbance was monitored at 405 nm and at room temperature for 10 min, and the absorbance was read every 15 s. Absorbance was corrected against the blank, and the slope of the linear region was determined to calculate the specific activity (ε_pNA_^31^ = 9500 M^–1^cm^–1^; *M*_Wtrypsin_:^32^ 24 kDa). Specific activity was plotted as a function of substrate concentration, and the curve was fit using the Michaelis–Menten formula and the Solver add-in from Microsoft Excel to yield the kinetic parameters.

## Results and Discussion

### BLC23O pH Optima in HEPES, Tris-HCl, and Sodium Phosphate

**Figure 2:**
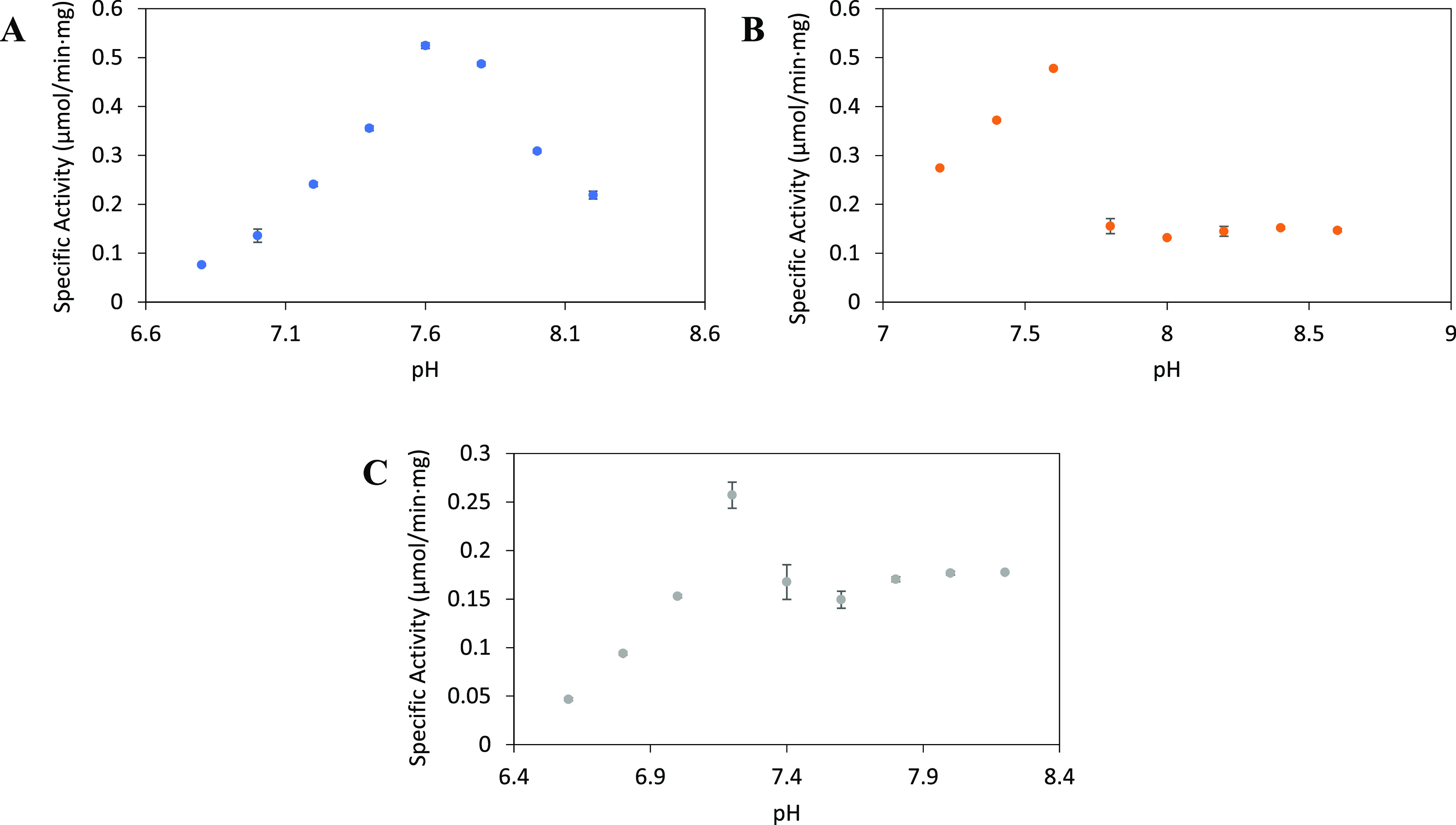
pH optima of BLC23O in HEPES (A), Tris-HCl (B), and in phosphate (C) buffers. Reactions contained 50 mM of the respective buffers, 1 mM 3-methylcatechol, 10 μM Mn^2+^ (except for reactions in phosphate that contained 100 μM Mn^2+^), and 18.6 μg/mL BLC23O. Absorbance was monitored at 388 nm for 60 min at 32.5 °C and read every 30 s. Reactions were performed in triplicate, and the absorbance was corrected against the blank. The initial linear slope was used to calculate the specific activity using the Beer–Lambert equation. Error bars represent the standard deviation.

The pH optima of BLC23O in each of the three buffers were identified. A pH range was chosen for each buffer according to its p*K*_a_, and BLC23O specific activity was determined at each pH point. In HEPES (Figure 2A), specific activity increased to a maximum at pH 7.6, and this was also observed in Tris-HCl (Figure 2B). However, the profiles of panels (A) and (B) were significantly different. While pH changes with HEPES buffer led to a bell-shaped curve, Tris-HCl yielded an immediate drop at pH 7.8. Crystallization studies showed that the active sites of EDOs have a 2-His-1-carboxylate motif that forms a shell around the metal cofactor.^33^ Sequence alignment of BLC23O with other EDOs suggested that the active site of the BLC23O enzyme may also contain a histidine residue, the side chain of which may interact with the Mn^2+^ ion. Since the buffering functional group for Tris-HCl is a primary amine (p*K*_a_= 8.1) and for histidine an imidazole group (p*K*_a_ = 7.6), when the pH increases in Tris-HCl buffer, both the amine and imidazole groups lost a proton. However, at higher pHs, the Mn^2+^ ion may leave the active site of the enzyme because it has more affinity for the NH_2_ group in the environmental buffer via a chelating effect. Therefore, the BLC23O enzyme demonstrated an abrupt activity drop between pH 7.6 and 7.8 in Tris-HCl buffer. This phenomenon is not observed in HEPES because HEPES does not have an amine group for buffering and therefore has a less chelating effect than Tris-HCl against the Mn^2+^ ion. In Na-phosphate buffer (Figure 2C), peak activity was recorded at pH 7.2 and higher pH led to similarly lower activity. This is possibly caused by the high affinity between Mn^2+^ and PO ^3–^ depleting the Mn^2+^ ion from the active site of the enzyme. These results suggested that different buffers significantly affected the activity profile and pH optimum of BLC23O.

### Buffer Impact on BLC23O Temperature Optimum

**Figure 3:**
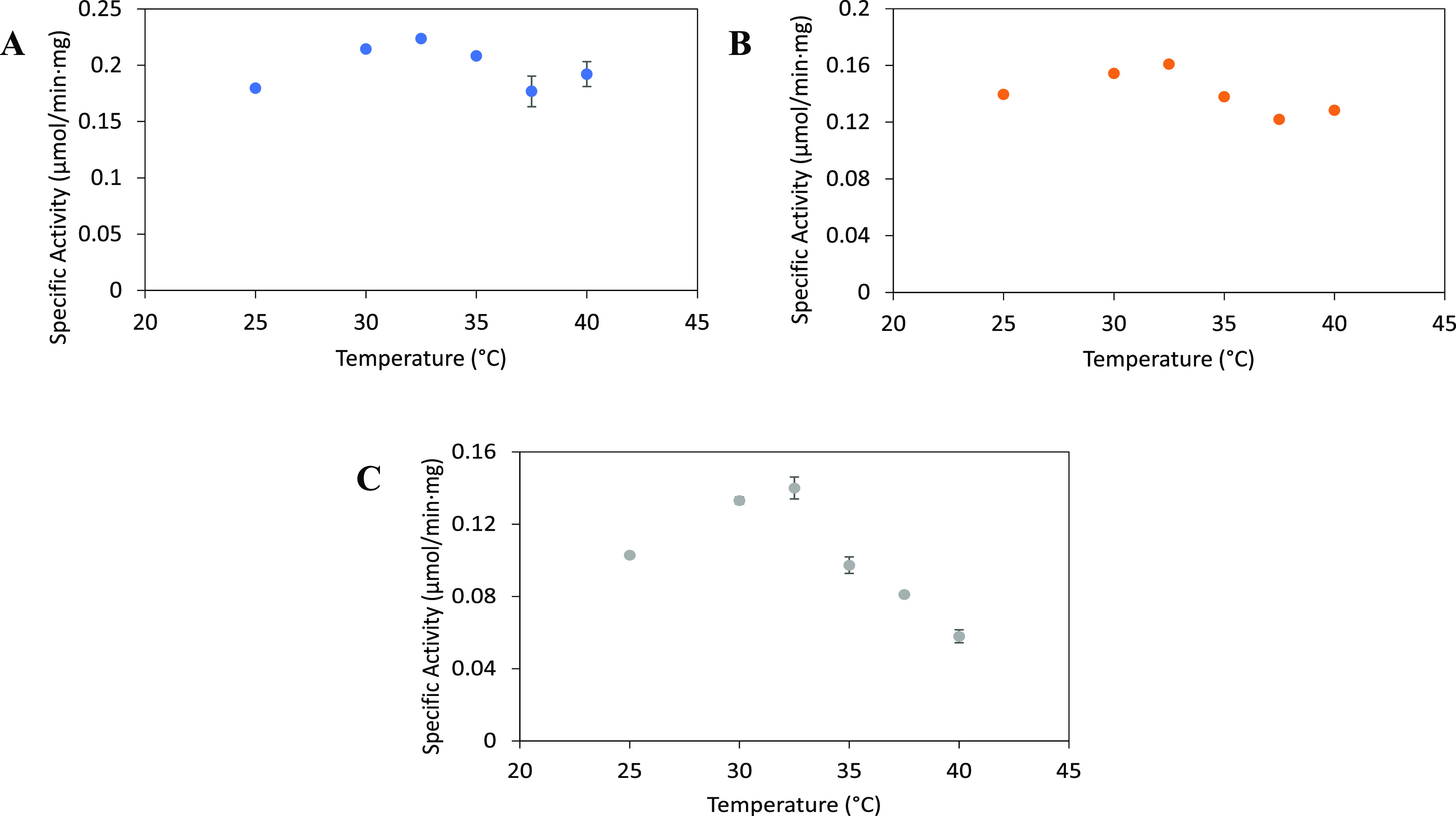
BLC23O temperature profile in HEPES (A), Tris-HCl (B), and phosphate (C) buffer. Reactions in HEPES were performed at pH 7.6, in Tris-HCl at pH 7.4, and in phosphate at pH 7.2. Each reaction contained 50 mM buffer, 1 mM 3-methylcatechol, 10 μM Mn^2+^ (100 μM Mn^2+^ in phosphate buffer), and 18.7 μg/mL BLC23O enzyme. Kinetic curves were collected at 388 nm for 60 min and read for every 30 s interval. The corrected absorbance against the blank was used to determine the slopes to calculate the specific activity under each condition.

BLC23O temperature optimum was obtained in each buffer at their individual optimal pH. In HEPES at pH 7.6 (Figure 3A), the optimal temperature obtained was 32.5 °C. The same temperature optimum was observed in Tris-HCl buffer at pH 7.4 (Figure 3B). In Na-phosphate buffer (pH 7.2), the temperature optimum was also recorded at 32.5 °C (Figure 3C). However, the activity profile at higher temperatures was much different compared to that of Tris-HCl and HEPES. Significantly decreased specific activity was observed. The specific activity decrease can signify either reduced product formation or quick product degradation. For instance, Fujiwara et al.^34^ reported that the product of C23O cleavage on 3-methylcatechol was unstable. Therefore, in the present study, it may be that certain combinations of buffer, pH, and temperatures led to an unstable product that is rapidly degraded, as shown in Figures 1B and 2C.

### Determination of BLC23O Kinetic Parameters

#### Determination of *K*_d_ for Manganese (Mn^2+^)

**Figure 4:**
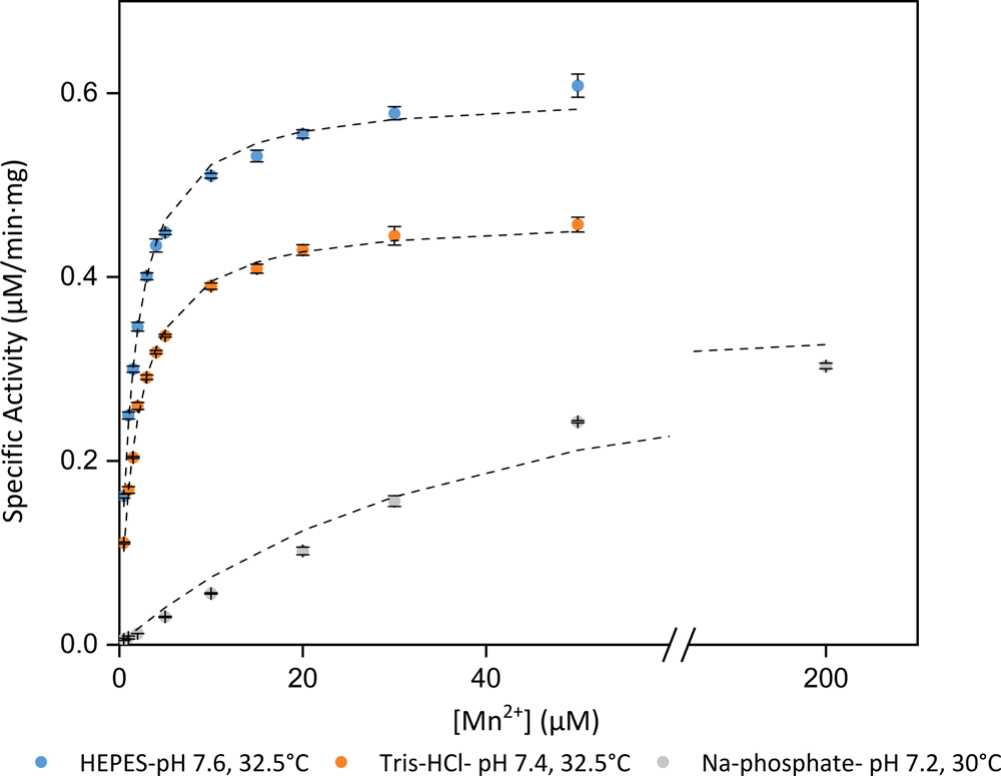
Dependence of BLC23O activity at varying concentrations of Mn^2+^ in the optimal conditions for HEPES (blue), Tris-HCl (orange), and Na-phosphate (gray). The 250 μL reactions (*n* = 3) contained 50 mM of the respective buffers, 1 mM 3-methylcatechol, 18.7 μg/mL BLC23O, and varying concentrations of MnCl_2_·4H_2_O. Reactions were initiated by the addition of 3-methylcatechol and were monitored at λ = 388 nm for 60 min. The specific activity was calculated by using the blank-corrected absorbance. The mean specific activity ± standard deviation is shown, and the dashed lines represent the specific activity curve model created with the Solver add-in from Microsoft Excel. An axis break is shown between 60 and 180 μM Mn^2+^. The graph was created by using OriginLab.

Determination of both the manganese dissociation constant *K*_d_ and the Michaelis–Menten kinetic parameters was performed under the same conditions in all three buffers (pH 7.4 and 32.5 °C) as well as under the established optimal conditions for each buffer (Table 1). The purpose of *K*_d_ determination was to identify the affinity of BLC23O for manganese and to compare this across different buffers. In addition, a suitable Mn^2+^ concentration for the Michaelis–Menten kinetic assay was determined. The *K*_d_, or the metal ion dissociation constant, was calculated according to Newman et al.^27,35^ and is analogous to *K*_m_ in that it is a measure of enzyme affinity for the metal cofactor. As such, a lower *K*_d_ represents a greater affinity, and the opposite is true for a higher *K*_d_. Under the set of optimal conditions previously established for each buffer (Figure 4: HEPES: pH 7.6, 32.5 °C; Tris-HCl: pH 7.4, 32.5 °C; and Na-phosphate: pH 7.2, 30 °C), it was observed that BLC23O exhibited the lowest *K*_d_ in HEPES at pH 7.6 (1.49 ± 0.05 μM), followed by Tris-HCl (1.79 ± 0.01 μM), and Na-phosphate buffer (44.24 ± 1.36 μM). Even though the first two buffers did not vary in *K*_d_ significantly, the third buffer showed a >20 times difference. The different *K*_d_ values obtained demonstrate that the affinity of the enzyme for manganese was significantly affected by the buffer identity. Moreover, the higher *K*_d_ in Na-phosphate buffer illustrated that this buffer lowered BLC23O manganese affinity of BLC23O compared to HEPES. Tris-HCl also lowered the manganese enzyme affinity but to a lesser extent.

**Table 1:** Kinetic Parameters *K*_d_, *K*_m_, *k*_cat_¸, and *k*_cat_/*K*_m_ for BLC23O in HEPES, Tris-HCl, and Phosphate Buffers under Different Experimental Conditions^a^

	*K*_d_ (μM)	*K*_m_ (mM)	*k*_cat_ (s^–1^)	*k*_cat_/*K*_m_(mM^–1^ s^–1^)
HEPES (pH 7.6)^b^	1.49 ± 0.05	0.54 ± 0.02	0.45 ± 0.01	0.84 ± 0.02
HEPES (pH 7.4)^c^	1.79 ± 0.02	0.48 ± 0.01	0.32 ± 0.01	0.67 ± 0.01
Tris-HCl (pH 7.4)^b^^c^	1.79 ± 0.01	0.71 ± 0.02	0.33 ± 0.00	0.47 ± 0.01
Na-Phosphate (pH 7.2)^b^	44.24 ± 1.36	0.76 ± 0.01	0.27 ± 0.00	0.36 ± 0.01
Na-Phosphate (pH 7.4)^c^	55.37 ± 3.65	0.24 ± 0.01	0.07 ± 0.01	0.28 ± 0.00

a Specific activity was calculated with the corrected slope obtained at A388nm. Kinetic curves were then modelled with the Michaelis–Menten formula and with the Excel Solver add-in. Error bars show the standard deviation between triplicates.

b Experiments performed under the optimal conditions for each buffer.

c Experiments performed under identical experimental conditions: 32.5°C and pH 7.4.

Another set of experiments was carried out to measure BLC23O activities under the same experimental conditions in all three buffers (pH 7.4 and 32.5 °C; Table 1) rather than the previously established optimal conditions. Under the same conditions, the *K*_d_ values obtained in both HEPES and Tris-HCl were not significantly different (*K*_d (H 7.4)_ = 1.79 ± 0.02 μM, *K*_d (T 7.4)_ = 1.79 ± 0.01 μM; *P* = 0.79), suggesting that the manganese affinity of the enzyme is the same in both buffers under identical conditions. Consistent with the previous results, under these conditions, the calculated *K*_d_ in phosphate buffer was 55.37 ± 3.65 μM, which is significantly higher than that in Tris-HCl and HEPES. This again suggested poor enzyme affinity for manganese. Based on the *K*_d_ values obtained, the Mn^2+^ concentration chosen for the Michaelis–Menten kinetic assays with HEPES and Tris-HCl buffer was 10 and 100 μM with Na-phosphate buffer.

#### Buffer Impact on Kinetic Constants *K*_m_, *k*_cat_ and *k*_cat_/*K*_m_

To determine the *K*_m_, *k*_cat_, and catalytic efficiency (*k*_cat_/*K*_m_) of BLC23O, kinetic assays were performed at different concentrations of 3-methylcatechol in the three buffers. The specific activity was graphed, and the curve was fitted to the Michaelis–Menten equation using the Solver add-in from Excel to obtain the kinetic parameters (Table 1). *K*_m_ is a measure of enzyme affinity for its substrate and is defined as the substrate concentration at which the rate of reaction is 50% of the maximum rate (*V*_max_). As a result, a low *K*_m_ corresponds to higher enzyme affinity, and the opposite is true for a high *K*_m_. *k*_cat_ is the turnover number of the enzyme or the number of catalytic events per second, and the *k*_cat_/*K*_m_ ratio defines the catalytic efficiency of the enzyme. Under the established optimal conditions for each buffer, it was observed that BLC23O exhibited the lowest *K*_m_ in the presence of HEPES (0.54 ± 0.02 mM) and therefore had higher substrate affinity compared to Tris-HCl and to Na-phosphate. Similarly, in terms of the turnover number (*k*_cat_) and the catalytic efficiency of BLC23O, the enzyme exhibited greater efficiency in HEPES (*k*_cat_= 0.45 ± 0.01 s^–1^; *k*_cat_/*K*_m_ = 0.84 ± 0.02 mM^–1^ s^–1^), followed by Tris-HCl, and last by phosphate. These results therefore show that buffer identity influenced the *k*_cat_, *K*_m_, and catalytic efficiency of metalloenzyme BLC23O.

**Figure 5:**
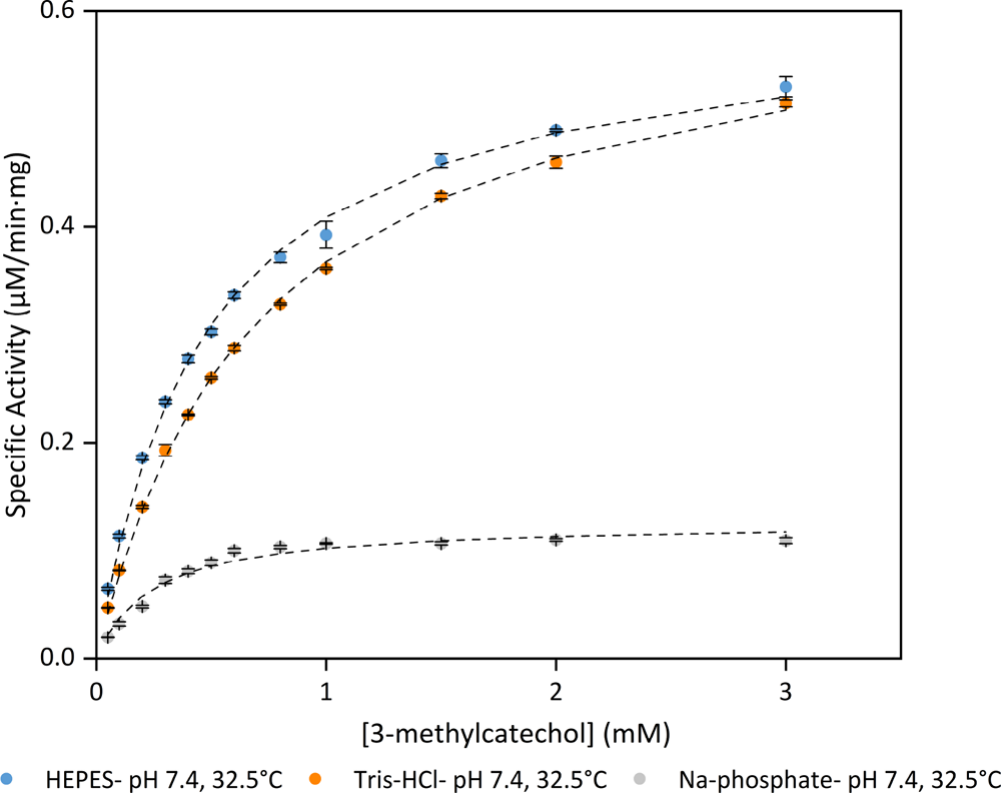
Kinetic curves of BLC23O in HEPES (orange), Tris-HCl (blue), and Na-phosphate (gray) under the same experimental conditions (pH 7.4 and 32.5 °C). The 250 μL reactions (*n* = 3) contained 50 mM of the respective buffers, varying concentrations of 3-methylcatechol, 18.7 μg/mL BLC23O, and MnCl_2_·4H_2_O (10 μM in HEPES and Tris-HCl and 100 μM in Na-phosphate). Reactions were initiated by the addition of 3-methylcatechol and were monitored at λ = 388 nm for 60 min. The specific activity was calculated using the blank-corrected absorbance. The mean specific activity ± standard deviation is shown, and the dashed lines represent the specific activity curve modeled using the Michaelis–Menten formula and the Solver add-in from Microsoft Excel. The graph was generated by using OriginLab.

The kinetics assay was also performed under the same conditions in all three buffers (pH 7.4 and 32.5 °C; Figure 5). BLC23O exhibited the highest substrate affinity in phosphate buffer (*K*_m (P 7.4)_ = 0.24 ± 0.01 mM), followed by HEPES and last by Tris-HCl. While BLC23O demonstrated the greatest *k*_cat_ in Tris-HCl (*k*_cat (T 7.4)_ = 0.33 ± 0.002 s^–1^), a 0.20 mM^–1^ s^–1^ higher catalytic efficiency was observed in the presence of HEPES compared to Tris-HCl. Phosphate buffer yielded both the lowest *k*_cat_ and catalytic efficiency of the three buffers. Once again, these results showed that even under the same experimental conditions, the identity of the buffer system impacts the kinetic parameters of BLC23O, thereby influencing the experimental results obtained. In addition, the observations again suggested that out of the three buffers, the enzyme exhibits greater catalytic efficiency in HEPES.

### Influence of Buffer on Catalytic Efficiency of BLC23O as a Factor of *K*_d_

**Figure 6:**
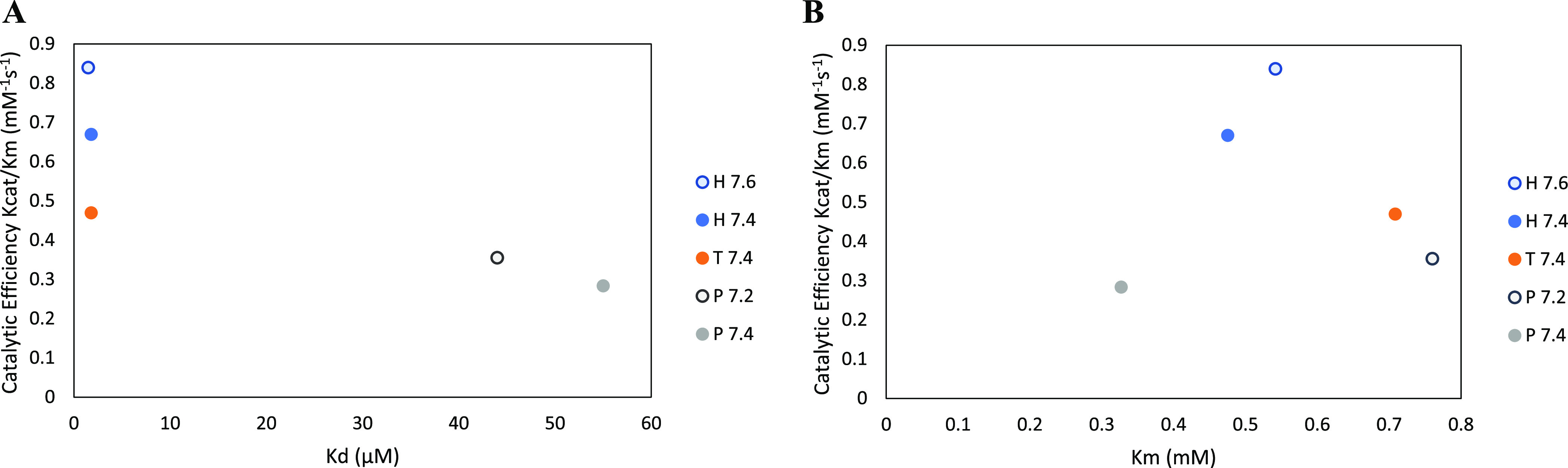
Catalytic efficiency of BLC23O in relation to *K*_d_ (enzyme affinity for metal ion cofactor: Mn^2+^) (A) and in relation to *K*_m_ (enzyme affinity for the substrate: 3-methylcatechol) (B) for each experimental condition.

To determine whether catalytic efficiency was correlated with either *K*_d_ or *K*_m_, catalytic efficiency was graphed either as a function of *K*_d_ (Figure 6A) or as a function of *K*_m_ (Figure 6B). From Figure 3A, illustrating the relationship between *k*_cat_/*K*_m_ and *K*_d_, it was observed that the catalytic efficiency decreased as *K*_d_ increased. As a result, the condition yielding the lowest *K*_d_ demonstrated the greatest catalytic efficiency (HEPES 7.6: *K*_d_ = 1.49 ± 0.05 μM; *k*_cat_/*K*_m_ = 0.84 ± 0.02 mM^–1^ s^–1^). On the other hand, phosphate buffer, which showed the highest *K*_d_ or the lowest manganese affinity, yielded the lowest catalytic efficiency (P 7.4: *K*_d_ = 55.37 ± 3.65 μM; *k*_cat_/*K*_m_ = 0.28 ± 0.00 mM^–1^ s^–1^). This negative correlation was not observed between catalytic efficiency and *K*_m_ (Figure 6B), as the condition yielding the lowest *K*_m_ (P 7.4), and therefore the highest substrate affinity, was also the one yielding the lowest catalytic efficiency. On the other hand, while HEPES at pH 7.6 resulted in the greatest catalytic efficiency, the *K*_m_ was in the middle (*K*_m_ = 0.54 ± 0.02 mM) between that of phosphate and Tris-HCl. These data therefore suggested that catalytic efficiency is most affected by BLC23O’s manganese affinity. As such, the effect of the buffer on manganese affinity, or *K*_d_, ultimately impacts the catalytic rate and catalytic efficiency of the enzyme.

### Influence of Buffers on *R. opacus* 1,2-catechol Dioxygenase Kinetic Parameters

In order to investigate whether the initial observations for BLC23O were valid for other metal-dependent enzymes, characterization of Fe^3+^-dependent *Ro*1,2-CTD activity was performed in the three buffers at pH 7.2 and at room temperature.^27^ The activity of the enzyme was graphed as a function of 3-methylcatechol concentrations, and the curve was fitted using the Michaelis–Menten equation and the Solver add-in from Microsoft Excel. The results (Table 2) showed that while BLC23O exhibited the lowest *k*_cat_ in HEPES (*k*_cat_= 0.64 ± 0.00 s^–1^), the *K*_m_ was also the lowest observed (1.80 ± 0.06 μM), indicating higher substrate affinity. Moreover, the catalytic efficiency in HEPES was the highest among all three buffers (0.36 ± 0.01 μM^–1^ s^–1^). On the other hand, the enzyme demonstrated the highest turnover rate in Tris-HCl (*k*_cat_ = 1.14 ± 0.01 s^–1^), despite having the highest *K*_m_ and thus the lowest substrate affinity (6.93 ± 0.26 μM). In this buffer, the catalytic efficiency of BLC23O was determined to be 0.17 ± 0.01 μM^–1^ s^–1^. Lastly, in sodium phosphate buffer, all three kinetic values were in between the other 2 buffers: the turnover number was 1.006 ± 0.006 s^–1^, while the *K*_m_ was 3.6 ± 0.1 μM and the catalytic efficiency was 0.28 ± 0.01 μM^–1^ s^–1^. Although in HEPES, the enzyme displays greater catalytic efficiency, the *Ro*1,2-CTD was only more efficient at low substrate concentrations, while at high substrate concentrations Tris-HCl favored greater specific activity. Once again, these results show that Fe^3+^-dependent *Ro*1,2-CTD has different kinetic parameters depending on the buffer, supporting the conclusion that buffer identity influences experimental results. As such, experimental results from different laboratories should not be compared when metalloenzymes are involved and when different buffers have been used. Accurate comparisons can only be conclusive when the same buffer system and experimental conditions are used.

**Table 2:** Kinetic Parameters *K*_m_, *k*_cat_, and *k*_cat_/*K*_m_ for *Ro*1,2-CTD in HEPES, Tris-HCl, and Phosphate Buffers^a^

	*K*_m_(μM)	*k*_cat_ (s^–1^)	*k*_cat_/*K*_m_ (μM^–1^ s^–1^)
HEPES	1.80 ± 0.06	0.64 ± 0.00	0.36 ± 0.01
Tris-HCl	6.93 ± 0.26	1.14 ± 0.01	0.17 ± 0.01
Na-phosphate	3.64 ± 0.11	1.01 ± 0.01	0.28 ± 0.01

a Reactions (n = 3) contained 50 mM buffer (pH 7.2) and 1.37 μg/mL *Ro* 1,2-CTD and was initiated with the addition of 3-methylcatechol to different final concentrations. Absorbance was monitored at room temperature and at 260 nm for a period of 30 min and read every 15 s. Absorbances were corrected against the blank, and the initial linear range slope was used to calculate the specific activity of the enzyme. The kinetic curve was modeled with the Michaelis–Menten equation to obtain the kinetic parameters. Error represents standard deviation from triplicates.

### Buffer Influence on Trypsin Kinetic Parameters

To determine whether buffer identity influences the activity of nonmetalloenzymes, trypsin from bovine pancreas was chosen as a model protein and characterized in the three buffers used in this study. Kinetic analysis was done in each buffer at pH 8.0 and room temperature. The specific activity was calculated and graphed as a function of BApNA concentrations to obtain kinetic parameters (Table 3). The *K*_m_ of trypsin observed in HEPES, Tris-HCl, and phosphate buffer was 3.14 ± 0.14, 3.07 ± 0.16, and 2.9 ± 0.02 mM, respectively. The negligible difference between the three *K*_m_ values suggested that buffer identity had little to no impact on enzyme affinity for BApNA. In addition, both the *k*_cat_ and the catalytic efficiency values observed in all three buffers showed a minimal difference. The kinetic characterization of trypsin showed that the kinetic values obtained in the three different buffers are comparable, suggesting that while buffer identity influences the activity of metalloenzymes, such as dioxygenases, it may not impact the activity of nonmetalloenzymes like trypsin.

**Table 3:** Kinetic Parameters *K*_m_,*k*_cat_¸, and*k*_cat_/*K*_m_ for Trypsin from Bovine Pancreas in HEPES, Tris-HCl, and Sodium Phosphate Buffer^a^

	*K*_m_ (mM)	*k*_cat_ (s^–1^)	*k*_cat_/*K*_m_ (mM^–1^ s^–1^)
HEPES	3.14 ± 0.14	1.51 ± 0.02	0.48 ± 0.01
Tris-HCl	3.07 ± 0.16	1.47 ± 0.02	0.48 ± 0.02
Na-phosphate	2.91 ± 0.02	1.53 ± 0.01	0.52 ± 0.00

a Reactions (*n* = 3) contained 100 mM buffer (pH 8.0) and 20 μg/mL trypsin and were initiated with the addition of BApNA. Absorbance was monitored for 10 min at 405 nm and at room temperature and corrected with the blank, and the specific activity was obtained (ε_pNA_= 9500 M^–1^cm^–1^). The kinetic curve was modeled with the Michaelis–Menten formula and the Solver add-in from Microsoft Excel to yield the kinetic parameters.

## Conclusions

To determine whether buffer identity influences enzyme activity, the characterization of three different enzymes has been performed in HEPES, Tris-HCl, and sodium phosphate buffers. Two of these enzymes are metalloenzymes: BLC23O and *Ro*1,2-CTD, and the results have shown that the kinetic parameters varied in different buffers. In contrast, the nonmetalloenzyme, trypsin, yields similar kinetic parameters independent of the buffer. Moreover, the negative correlation between the catalytic efficiency of BLC23O and the *K*_d_ suggests that different buffers affect the catalytic efficiency and the activity of a metalloenzyme through their impact on the enzyme metal affinity. Taken together, these results suggest that while buffer identity impacts the activity of metal-dependent enzymes, the activities of the tested nonmetalloenzymes are not affected. A broader range of enzymes should be tested in the future to validate, in particular, the relationship between buffers and nonmetalloenzymes. Review of the literature reveals that reaction buffers may influence the activity of certain nonmetalloenzymes by reacting with protein substrates or by acting as either competitive^36^ or noncompetitive inhibitors. Additionally, the ionic strength of buffers has also been observed to affect enzyme activity^37^ by altering their structural characteristics and solubility.^38^ Therefore, we suggest that buffer selection be optimized for any novel enzyme characterization and activity analyses. Furthermore, the same buffer system should be utilized when comparing experimental results across papers as much as possible.

## Supporting Information Available

SDS-PAGE analysis for the purification of BLC23O overexpressed in*E. coli* BL21 (DE3) cells and BLC23O quantification using the Bradford method (PDF)
