# Supplementary material for: Assay Development for Metal-Dependent Enzymes—Influence of Reaction Buffers on Activities and Kinetic Characteristics
Source: ACS Omega. 2023 Oct 18;8(43):40119–27. doi: 10.1021/acsomega.3c02835 (PMC10620931; doi:10.1021/acsomega.3c02835)
Supplement: Supplementary file 1 — ao3c02835_si_001.pdf [file ao3c02835_si_001.pdf]

Assay Development for metal-dependent enzymes-influence of reaction buffers on activities and kinetic characteristics

Natalia Forero[1], Chengsong Liu[2], Sami George Sabbah[3], Michele C. Loewen[2], Trent Chunzhong Yang[2]*

1. Department of Chemistry and Biomolecular Sciences, University of Ottawa; 2. Aquatic and Crop Resource Development Research Centre, National Research Council, Ottawa, ON, Canada; 3. Department of Medicine, University of Ottawa, Ottawa, ON, Canada

SDS-PAGE

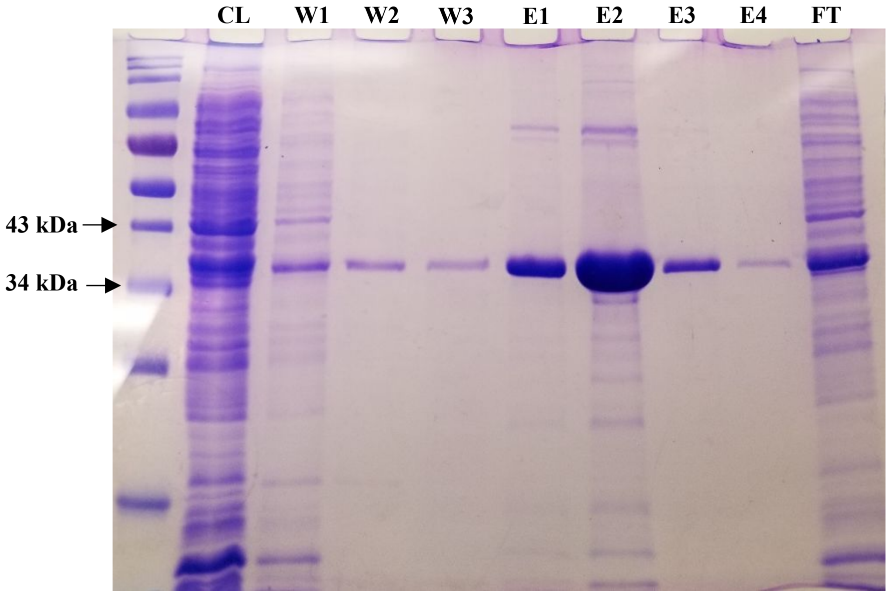

Figure S1. Ni-NTA purification results of BLC23O sample. Samples were run on a 12% acrylamide gel at 80V for 30 minutes and then at 100V for 20 minutes. The ladder was added in the first well, followed by the cell lysate (CL). Washes are denoted as **W**, and elutions as **E**; the flowthrough was added in the last well.

Bradford assay standard curve

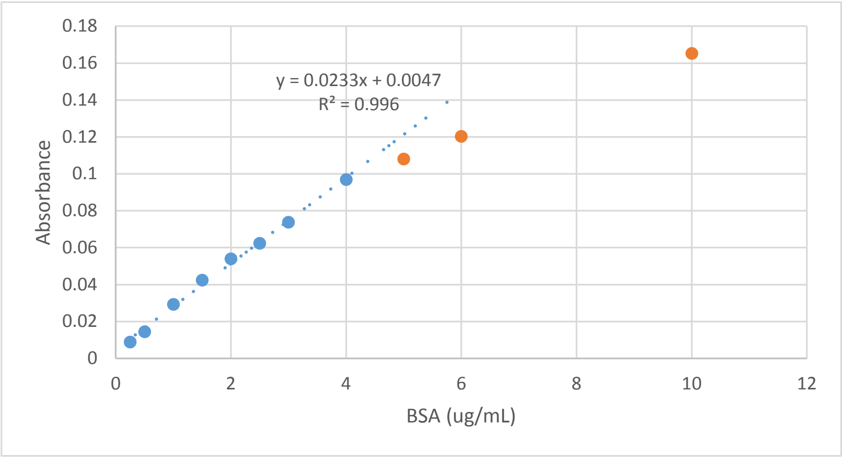

Figure S2. Standard curve for the Bradford Assay by targeting specific BSA concentrations. BSA was used as the protein standard, and the well reaction volume was 200uL. 5x Bradford reagent was added into the well, and the absorbance was read at 595nm. Line of best fit given is y= 0.0233x + 0.0047 (R^2=0.996).
